# Strength of forelimb lateralization predicts motor errors in an insect

**DOI:** 10.1098/rsbl.2016.0547

**Published:** 2016-09

**Authors:** Adrian T. A. Bell, Jeremy E. Niven

**Affiliations:** School of Life Sciences and Centre for Computational Neuroscience and Robotics, University of Sussex, Falmer, Brighton BN1 9QG, UK

**Keywords:** handedness, locust, limb control, reaching, motor control

## Abstract

Lateralized behaviours are widespread in both vertebrates and invertebrates, suggesting that lateralization is advantageous. Yet evidence demonstrating proximate or ultimate advantages remains scarce, particularly in invertebrates or in species with individual-level lateralization. Desert locusts (*Schistocerca gregaria*) are biased in the forelimb they use to perform targeted reaching across a gap. The forelimb and strength of this bias differed among individuals, indicative of individual-level lateralization. Here we show that strongly biased locusts perform better during gap-crossing, making fewer errors with their preferred forelimb. The number of targeting errors locusts make negatively correlates with the strength of forelimb lateralization. This provides evidence that stronger lateralization confers an advantage in terms of improved motor control in an invertebrate with individual-level lateralization.

## 1. Introduction

Lateralization, the functional and/or structural specialization of either side of the brain/body, is widespread among vertebrate and invertebrate bilaterians alike [1,2]. Lateralization can occur in perception, motor control and/or cognitive processing yet the potential advantages that such laterality confers, and how it evolved, remain unclear. Studies have demonstrated differences among individuals in the strength of lateralization on particular tasks, and shown that more lateralized animals typically out-compete their non-lateralized counterparts [3–7]. However, these studies have focused on vertebrates, many of which show population-level lateralization in which unequal proportions of right- and left-biased individuals coexist [1].

Such population-level lateralization is often proposed to have evolved from individual-level lateralization, in which there is no obvious mode in the strength and direction of the bias [1]. In turn, individual-level lateralization is supposed to confer an advantage in terms of proximal performance that improves ultimate success, only coalescing into population-level lateralization when the benefits of cooperativity outweigh the costs of predictability [8,9].

Locusts offer the possibility of assessing the advantage of lateralization in a species that shows individual-level lateralization: while performing visually targeted reaching their forelimb use is lateralized [10,11]. Consequently, individual locusts may be strongly or weakly lateralized in terms of forelimb use. We made use of this variation to determine whether the strength of individual lateralization confers an advantage in performance by assessing the accuracy of targeted forelimb placement during gap-crossing. We show that more strongly lateralized locusts are less prone to making errors than those with weaker biases demonstrating that, even in the absence of population-level handedness, stronger lateralization confers an advantage in terms of motor control in an invertebrate.

## 2. Material and methods

### (a) Animals

Fifth-instar desert locusts (*Schistocerca gregaria*, Forskål 1775) were selected at random from a crowded colony maintained throughout their final moult at the School of Life Sciences in heated holding tanks (24°C) and given wheat germ, grass and water from which they fed ad libitum. The locusts remained in the tanks except during testing. Only animals with intact eyes, limbs and antennae were selected for experiments. Individual locusts were identified by making a small unique cut in their wings.

### (b) Experimental arena and platform

Individual locusts were tested at 23°C in a rectangular white Perspex arena (800 × 600 × 600 mm) lined with cardboard [11]. A hole in the arena wall (60 mm diameter) permitted filming of the locusts, while an identical hole in the opposite wall containing a black disc maintained symmetry. Two horizontal platforms (150 × 50 × 20 mm) constructed from Perspex were placed opposite one another 25 mm apart in the centre of the arena [11]. The platform to which each locust crossed was elevated by 5 mm with an edge marked with black acrylic paint. The horizontal surface of each platform was covered with white paper to allow it to be cleaned easily. A black cardboard rectangle (60 × 250 mm), attractive to locusts, was placed at the end of this platform.

### (c) Testing

Individual locusts were tested for limb preference over 21–30 days. Each locust experienced only one gap-crossing trial before being returned to a holding tank to minimize task familiarity. Each locust was placed 80 mm from the gap, on the platform opposite the black cardboard rectangle. Filming began after the locust started to walk and continued until it crossed to the opposite platform. On half the trails the locusts crossed from right to left, while on the other half they crossed left to right.

### (d) Video analysis

The gap-crossing trials were recorded with a video camera (SONY Handycam HDR-CX105E) fitted with a wide-angle lens positioned parallel to the horizontal plane of the gap. Videos were saved and analysed offline.

### (e) Statistical analysis

The distribution of the forelimb use during reaching was tested for deviation from the expected binomial distribution (*p* = 0.5). Classes in which expected values were less than 3 were amalgamated with the adjacent classes. A *G*-test for goodness of fit to the intrinsic hypothesis was used with William's adjustment (*G*_adj_) applied [12]. A two-tailed exact binomial test was used to determine whether individual locusts deviated from the expected binomial distribution (*p* = 0.5). An independent samples *t*-test was conducted to compare the mean number of missed reaches between strongly and weakly biased individuals. A Spearman's rank correlation was used to test for a significant relationship between the strength of individual handedness and the number of reaching errors.

## 3. Results

**Figure 1.**
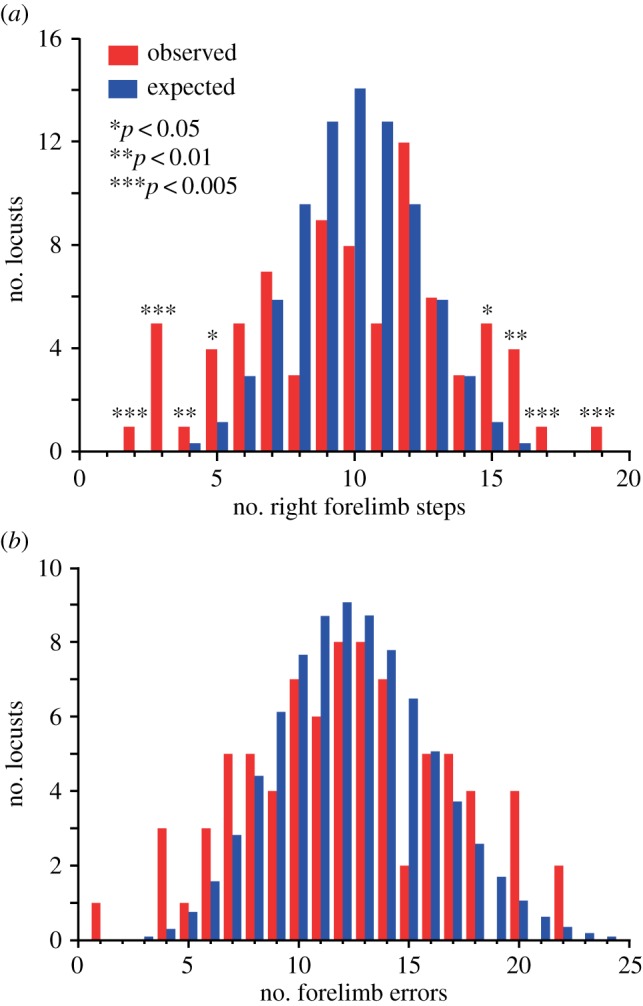
(*a*). Frequency distributions of movements initiated by the right forelimb (red) compared with the expected binomial distribution (*p* = 0.5) (blue). Asterisks indicate significant deviations from the binomial distribution determined by exact binomial tests. (*b*) Frequency distribution of reaching errors made by locusts (red) compared with the expected Poisson distribution (blue) (*N* = 80, *n* = 20).

Each locust (*N* = 80) performed 20 trials during which they had to reach across a gap in the platform on which they walked using either forelimb. The distribution of the locusts' forelimb use deviated significantly from the binomial expectation (*G*-test, *G*_adj_ = 42.38, 7 d.f., *p* < 0.005, *N* = 80), with individual locusts differing significantly in the strength and direction of their bias (figure 1*a*; electronic supplementary material, table S1). This confirmed that locusts possess an individual-level bias in forelimb use during targeted reaching with no consistent bias among the population towards the right or left forelimb [11].

When reaching across the gap with one forelimb towards the opposite platform, locusts occasionally made reaching errors missing the opposite side of the platform (electronic supplementary material, figure S1). During these errors, the forelimb swept down into the gap without contacting the opposite platform, before being re-targeted to the platform. To test whether they were independent, we compared the observed distribution of reaching errors against a Poisson distribution with the same mean (figure 1*b*). The observed and expected distributions did not differ significantly (*G*-test, *G*_adj_ = 15.01, 11 d.f., *p* > 0.05; *N* = 80, *n* = 20), suggesting that an error on one trial is independent of errors in previous or subsequent trials.

**Figure 2.**
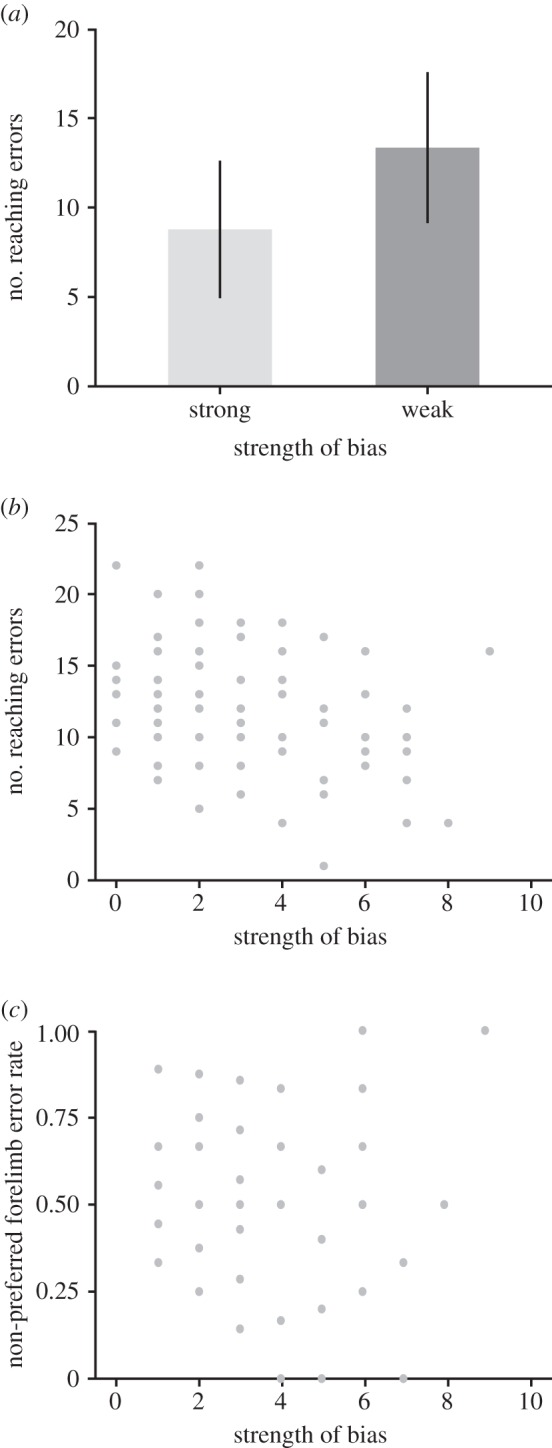
Strong forelimb preference is advantageous while gap-crossing. (*a*). Strongly biased individuals make fewer reaching errors than weakly biased individuals while gap-crossing. Error bars indicate the standard error (*N* = 80, *n* = 20). (*b*). The strength of an individual's bias is inversely related to the number of reaching errors (*N* = 80, *n* = 20). (*c*). There is no relationship between strength of bias and error rate in the non-preferred forelimb (*N* = 72, *n* = 20).

We compared the numbers of errors made by strongly biased locusts (significant exact binomial test) with those made by weakly biased locusts (non-significant exact binomial test; electronic supplementary material, table S1) to assess whether they differed in the number of forelimb errors they made. The 22 strongly biased locusts made on average 8.77 errors (s.d. = 3.84, *N* = 58), significantly fewer than the 58 weakly biased locusts (mean = 13.34, s.d. = 4.22, *N* = 22) (independent samples *t*-test; *t* = 4.43, 78 d.f., *p* = 0.00003) (figure 2*a*). This suggests that being strongly biased improves forelimb placement accuracy.

We then determined whether the strength of the locusts' forelimb bias was correlated with the number of reaching errors they made. The strength of forelimb bias was based on preferred forelimb usage; zero was assigned to locusts that crossed with their right forelimb in 10 of 20 trials, while 10 was assigned to those that crossed with their right forelimb in either 20 or 0 of 20 trials. We found a significant negative correlation between strength of bias and number of reaching errors (Spearman's rank-order correlation; *ρ* = −0.404, *N* = 80, 78 d.f., *p* = 0.0002; figure 2*b*), which suggests that the strength of the bias is related to the accuracy of forelimb placement.

Even strongly biased locusts occasionally reach across the gap using the non-preferred limb. If the advantage conferred by a stronger bias is specific to the preferred limb then, when using the non-preferred limb, strongly biased locusts should be no more accurate than their weakly biased counterparts. As expected, there was no correlation between strength of bias and number of reaching errors in the non-preferred forelimb (Spearman's rank-order correlation; *ρ* = −0.153, *N* = 72, 70 d.f., *p* = 0.199; figure 2*c*). This suggests that the strength of limb preference affects the error rate of the preferred limb but not the non-preferred limb.

## 4. Discussion

Individual desert locusts differ in the strength of lateralization they display while making visually targeted forelimb movements with no particular bias to left or right across the population, confirming earlier reports from far smaller cohorts [11]. While making visually targeted forelimb movements, locusts occasionally make errors, missing their target [10]. We showed that the number of these errors is correlated with the strength of a locust's lateralization, demonstrating that in a species with individual-level lateralization, stronger lateralization improves motor control and reduces errors, conferring a direct advantage.

Although lateralized animals have been shown to out-compete non-lateralized animals in previous studies that focused on vertebrates [3–7], our results demonstrate this in an invertebrate. Our findings fit with the hypothesis that individual-level lateralization has advantages for individuals [8], even in species lacking any obvious lateralization of their central nervous system. However, it also emphasizes that there is no necessary progression from individual- to population-level lateralization, implying that specific combinations of selective pressures can ensure species retain individual-level lateralization or that it coalesces into population-level handedness.

Forelimb reaching in locusts and other grasshoppers is targeted by visual inputs primarily from the ipsilateral eye, with seemingly little contribution from the contralateral eye [10,13]. This suggests there is a substantial separation of descending visuomotor pathways, which may allow specialization that is advantageous because it reduces the numbers of neurons involved in a reach. Lateralization within the visual system may also contribute to a bias in forelimb use. Experimental removal of vision from one compound eye in grasshoppers can alter forelimb use in a visually targeted reaching task [10,13], suggesting that visual bias can play a substantial role in forelimb selection. Moreover, inputs to the compound eyes of honeybees differ in their contribution to classical conditioning [14], raising the possibility that inputs from compound eyes could be specialized for specific tasks.

Specialization of motor circuits and mechanosensory reflexes has also been proposed to exist within the prothoracic ganglion of locusts, which controls forelimb movements [15,16], suggesting that particular limbs may be favoured in particular motor tasks. Initial preferences in forelimb use, possibly in earlier instars, could be reinforced by experience. Locusts, like cockroaches [17], are capable of motor learning at the level of single ganglia raising the possibility that lateralization of forelimb reaching arises through experience allowing circuits to be refined for a specific task.

The retention of weakly lateralized locusts suggests there are costs opposing the benefits associated with strong lateralization. One potential cost is predictability because competitors and/or predators can exploit predictable movements and/or decisions [8,9]. In the case of gregarious desert locusts, there may be fierce competition within a swarm, individuals being cannibalized by other locusts [18]. Consequently, there may be considerable selective pressure to avoid predictability, maintaining individual-level lateralization.

## Supplementary Material

Individual stepping biases

## Supplementary Material

Trajectories of forelimb reaches and errors

## Data accessibility

All supporting data can be found in the electronic supplementary material, tables.
