# Supplementary material for: Strength of forelimb lateralization predicts motor errors in an insect
Source: Biol Lett. 2016 Sep;12(9):20160547. doi: 10.1098/rsbl.2016.0547 (PMC5046935; doi:10.1098/rsbl.2016.0547)
Supplement: Individual stepping biases [file rsbl20160547supp1.docx]

Strength of forelimb lateralisation predicts motor errors in an insect

Adrian T. A. Bell, Jeremy E. Niven

School of Life Sciences and Centre for Computational Neuroscience and Robotics, University of Sussex, Falmer, Brighton BN1 9QG, United Kingdom

Individual stepping biases in locusts

**Table S1.** Binomial Exact tests of goodness-of-fit for steps up to opposite platform (N=80, n=20). Significant differences are denoted with asterisks (**P*<0.05; ***P*<0.01; ****P*<0.005)**.**

| **Locust** | **Right Forelimb Steps** | **2-tailed probability** |
| --- | --- | --- |
| 1 | 11 | 0.664 |
| 2 | 4 | 0.0072** |
| 3 | 16 | 0.0072** |
| 4 | 2 | 0.000221*** |
| 5 | 14 | 0.0784 |
| 6 | 12 | 0.383 |
| 7 | 3 | 0.00149*** |
| 8 | 15 | 0.0266* |
| 9 | 14 | 0.0784 |
| 10 | 11 | 0.664 |
| 11 | 9 | 0.664 |
| 12 | 13 | 0.189 |
| 13 | 11 | 0.664 |
| 14 | 7 | 0.189 |
| 15 | 13 | 0.189 |
| 16 | 6 | 0.0784 |
| 17 | 3 | 0.00149*** |
| 18 | 12 | 0.383 |
| 19 | 5 | 0.0266* |
| 20 | 7 | 0.189 |
| 21 | 11 | 0.664 |
| 22 | 13 | 0.189 |
| 23 | 7 | 0.189 |
| 24 | 5 | 0.0266* |
| 25 | 6 | 0.0784 |
| 26 | 10 | 1 |
| 27 | 9 | 0.664 |
| 28 | 10 | 1 |
| 29 | 8 | 0.383 |
| 30 | 19 | 0.000021*** |
| 31 | 11 | 0.664 |
| 32 | 13 | 0.189 |
| 33 | 12 | 0.383 |
| 34 | 6 | 0.0784 |
| 35 | 9 | 0.664 |
| 36 | 7 | 0.189 |
| 37 | 9 | 0.664 |
| 38 | 15 | 0.0266* |
| 39 | 12 | 0.383 |
| 40 | 9 | 0.664 |

| 41 | 5 | 0.0266* |
| --- | --- | --- |
| 42 | 5 | 0.0266* |
| 43 | 4 | 0.0072* |
| 44 | 8 | 0.383 |
| 45 | 17 | 0.00149*** |
| 46 | 12 | 0.383 |
| 47 | 4 | 0.0072** |
| 48 | 10 | 1 |
| 49 | 10 | 1 |
| 50 | 13 | 0.189 |
| 51 | 7 | 0.189 |
| 52 | 11 | 0.664 |
| 53 | 13 | 0.189 |
| 54 | 15 | 0.0266* |
| 55 | 10 | 1 |
| 56 | 10 | 1 |
| 57 | 8 | 0.383 |
| 58 | 10 | 1 |
| 59 | 17 | 0.00149*** |
| 60 | 12 | 0.383 |
| 61 | 13 | 0.189 |
| 62 | 4 | 0.0072** |
| 63 | 8 | 0.383 |
| 64 | 17 | 0.00149*** |
| 65 | 11 | 0.664 |
| 66 | 8 | 0.383 |
| 67 | 5 | 0.0266* |
| 68 | 7 | 0.189 |
| 69 | 8 | 0.383 |
| 70 | 3 | 0.00149*** |
| 71 | 14 | 0.0784 |
| 72 | 11 | 0.664 |
| 73 | 11 | 0.664 |
| 74 | 9 | 0.664 |
| 75 | 8 | 0.383 |
| 76 | 15 | 0.0266* |
| 77 | 10 | 1 |
| 78 | 14 | 0.0784 |
| 79 | 8 | 0.383 |
| 80 | 6 | 0.0784 |
