# Supplementary figures and images for: Strength of forelimb lateralization predicts motor errors in an insect
Source: Biol Lett. 2016 Sep;12(9):20160547. doi: 10.1098/rsbl.2016.0547 (PMC5046935; doi:10.1098/rsbl.2016.0547)

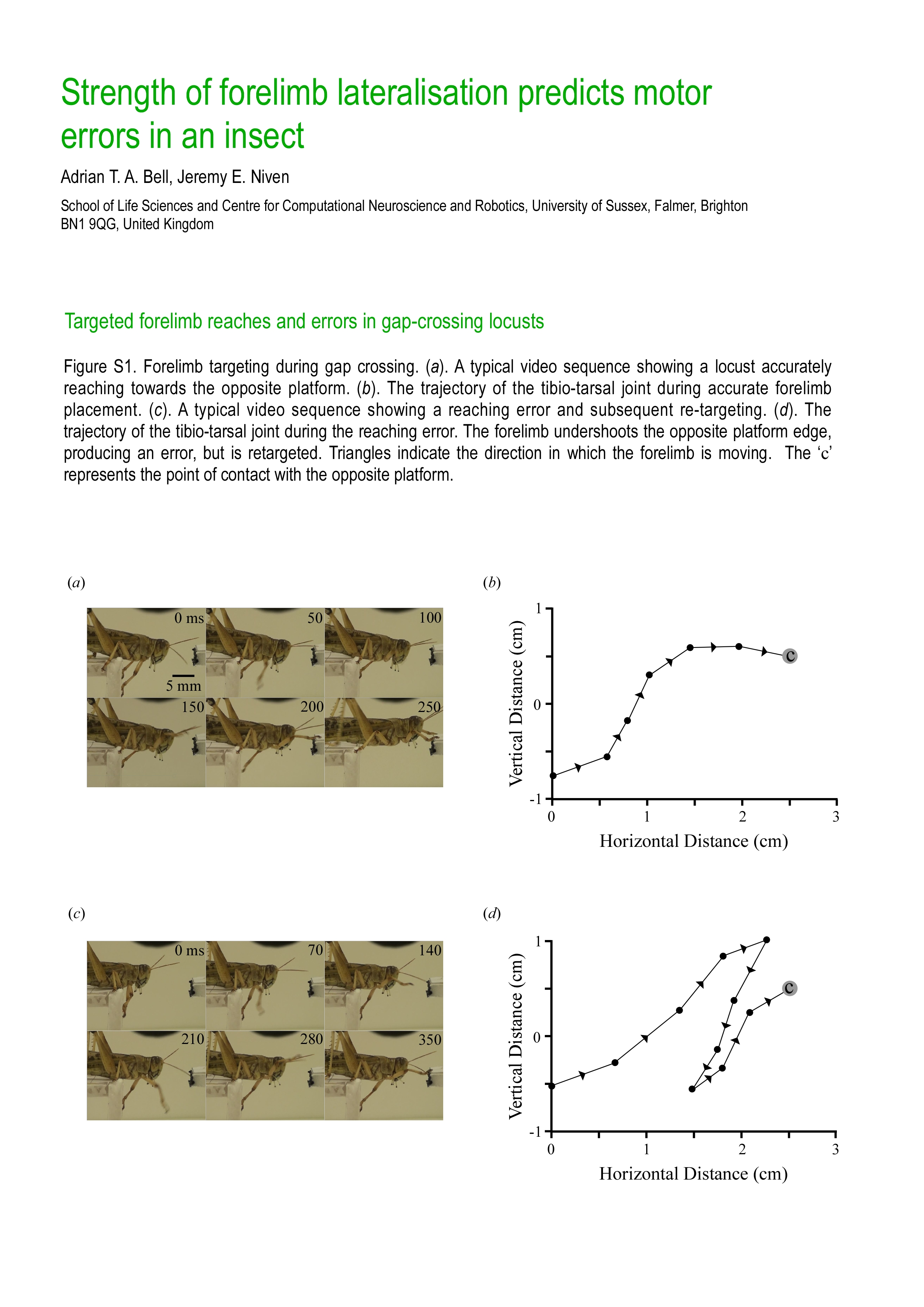

Supplement: Trajectories of forelimb reaches and errors [file rsbl20160547supp2.jpg]
